# Prognostic marker C3AR1 is associated with ovarian cancer cell proliferation and immunosuppression in the tumor microenvironment

**DOI:** 10.1186/s13048-023-01140-2

**Published:** 2023-04-01

**Authors:** Jinfa Huang, Lei Zhou, Kaixian Deng

**Affiliations:** Department of Gynecology, Shunde Hospital, Southern Medical University, The First People’s Hospital of Shunde), Foshan, 528308 Guangdong China

**Keywords:** Immune infiltration, C3AR1, Ovarian cancer, Tumor microenvironment, Immunotherapy

## Abstract

**Background:**

C3AR1 was reported in driving tumor immunity in multiple cancers. However, its roles in ovarian cancer remain unclear. This study aims to determine role of C3AR1 in prognosis and regulating tumor infiltrating immune cells of ovarian cancer (OC).

**Materials and methods:**

The expression, prognosis and clinical data related to C3AR1 were collected from public databases such as The Cancer Genome Atlas (TCGA), Human Protein Atlas (HPA) and Clinical Proteomics Tumor Analysis Alliance (CPTAC), and further analyze their relationship with immune infiltration. Immunohistochemistry verified the expression of C3AR1 in ovarian cancer and control tissues. C3AR1 was forced expressed in SKOV3 cells by plasmid transfection, and verified by qRT-PCR and Western blot. Cell proliferation were evaluated by EdU assay.

**Results:**

Bioinformatics analysis (TCGA, CPTAC) and immunohistochemical staining of clinical samples confirmed higher C3AR1 expression in ovarian cancer than that in normal tissues. High C3AR1 expression predicted adverse clinical outcomes. KEGG and GO analysis showed that the biological processes of C3AR1 in ovarian cancer are mainly involved in T cell activation, cytokine and chemokine activation. C3AR1 expression was positively correlated with chemokines and their receptors in the tumor microenvironment, such as CCR1(R = 0.83), IL10RA (R = 0.92), and INFG (R = 0.74). In addition, increased C3AR1 expression predicted more infiltration of tumor-associated macrophages, dendritic cell and CD8 + T cell. Some important m6A regulators, such as IGF2BP2, ALKBH5, IGFBP3 and METL14, are significantly positively or negatively correlated with C3AR1. Finally, overexpression of C3AR1 significantly increased proliferation of SKOV3 cells.

**Conclusion:**

In summary, our study suggested that C3AR1 is associated with the prognosis and immune cell infiltration of ovarian cancer, and is a promising immunotherapeutic target.

**Supplementary Information:**

The online version contains supplementary material available at 10.1186/s13048-023-01140-2.

## Introduction

Ovarian cancer (OC) is a highly aggressive malignant tumor, ranking fifth in the cancer-related death in [1]. Patients with ovarian cancer have a poor prognosis, more than half of them having a survival time less than 5 [2]. 90% of ovarian cancer are of epithelial origin, while the others are non-epithelial. Epithelial ovarian cancer includes many histological types, with respectively specific molecular changes, clinical behaviours, and treatment outcomes. Germ cell tumors and sex cord-stromal tumors are the main non-epithelial ovarian cancer. Germ cell tumors most often occur among younger women of childbearing age with good prognosis. Sex cord-stromal tumours affect all age groups, diagnosed with early stage, indolent [3]. In addition, mutations in DNA repair pathways increase the risk of chemotherapy resistance.The synthetic lethality of poly (ADP-ribose) polymerase (PARP) inhibitors is directed against BRCA mutations, which can be novel therapeutic targets for epithelial ovarian cancer treatment. Several PARP inhibitors, including olaparib, rucaparib, and niraparib, gained clinical approval from FDA and/or EMA for the treatment epithelial ovarian cancer. Olaparib, rucaparib, and niraparib approximately are more efficient than veliparib in trapping [4]. FIGO stage remains the most appropriate indicator for predicting the prognosis of OC, but it frequently fails to accurately predict the prognosis of a specific individual, as the survival of patients with the same disease stage varies [5]. Therefore, identifying biomarkers related to high-risk patients with low survival rates is of great significance. Proteomics technologies and tools have made it possible to reveal molecular events and the proteomic characterization involved in tumor development. At this point, proteomic profiling of ovarian cancer, as well as their adaptive responses to therapy, can help develop new therapeutic strategies for reducing drug resistance and improving patient [4].

Ovarian cancer cells evade immune surveillance and achieve tumor proliferation by shaping a highly immunosuppressive tumor microenvironment (TME)[6]. According to reports, tumor-associated macrophages (TAM), regulatory T cells (Treg), myeloid-derived suppressor cells (MDSC), and tumor-associated dendritic cells (tDC) together make suppressive immune [7]. TAMs with immunosuppressive M2 phenotype account for the majority of tumor microenvironments, and stimulate cancer [8], metastasis, angiogenesis, immune [9], and tumor [10]. Therefore, exploring new strategies to identify molecules targets of the tumor microenvironment (TME), especially TAM, may improve the therapeutic effect and 5-year survival rate of OC patients.

Existence of cancer is often accompanied by changes in the complement system. On the one hand, due to its water-soluble properties, complement can often infiltrate into deeper tumors faster than immune cells. It recruits leukocytes through anaphylactoxin, opsonin and lysis, promotes tumor lysis and induces the release of pro-inflammatory mediators to enhance the anti-cancer [11]. On the other hand, the complement system itself has the functions of angiogenesis, promotion of cell invasion and tissue [12]. Therefore, complement also has a tumor-promoting effect. The encoding gene of C3AR1 (C3a anaphylatoxin chemotactic receptor) locates on chromosome 12 and is also known as AZ3B; C3R1 and C3AR. C3AR1 belongs to the G protein coupled receptor 1 family. Accumulating evidence confirms the role of C3AR1 in cancer. Increased C3AR1 suggests poor prognosis in patients with gastric adenocarcinoma, and leads to an increase in suppressive tumor immune cell [13]. Pan-cancer analysis found that copy number variation and gene methylation are the main reasons for the increased expression of C3AR1 and are related to the dysfunctional T cell phenotype, which leads to tumor immune evasion and reduces the effect of [14]. Studies have shown that ovarian cancer patients higher C3 level tend to have a shorter overall [15]. C3 deficiency inhibits the formation of ovarian tumors in mice. In addition, down-regulating C3 expression reduces the growth rate of tumors in mouse ovarian cancer [16]. However, the prognostic value of C3AR1 and its possible mechanism in ovarian cancer remains unclear. In our current research, we use comprehensive bioinformatics analysis to explore the expression and clinical value of C3AR1 in OC, and further explore its regulatory network and its role in shaping the tumor immune microenvironment.

## Materials and methods

### Increased C3AR1 in OC and its clinical significance

The transcription levels of C3AR1 in pan-cancers were explored in the GEPIA (http://gepia.cancer-pku.cn/) database. Protein expression level of C3AR1 were investigated in the Clinical Proteomics Tumor Analysis Alliance (CPTAC) dataset and the HPA database (https://www.proteinatlas.org/). C3AR1 expression in different ovarian cancer cells were detected in CCLE (https://portals.broadinstitute.org/ccle). In addition, correlations between C3AR1 and the clinicopathological characteristics of OC patients is explored in TCGA database. Prognostic value of C3AR1 was analyzed in the Kaplan Meier plotter (http://kmplot.com/).

### Functional enrichment analysis of C3AR1 co-expression genes and differentially expressed genes

Co-expressed genes of C3AR1 were investigated by the R software and the Spearson correlation coefficient test. Genes with |R|≥0.6 and the p < 0.05 are considered as C3AR1 co-expressed. OC patients in the TCGA database was divided into two groups based on median C3AR1 expression. The differentially expressed genes (DEGs) were analyzed using the DESeq2.0 package, and criteria was |Log2FC|>2 and p < 0.05. Functional pathway analysis of GO and KEGG was conducted on DEGs and co-expressed genes individually, and visualized by the ggplot2 software package.

### Correlation between C3AR1 and tumor immune infiltration

To explore the role of C3AR1 in regulating immune microenvironment of OC, we analyzed the relationship between the expression of C3AR1 and B cells, CD4 + T cells, CD8 + T cells, macrophages, neutrophil cell and dendritic cell infiltration in the TIMER database (www.cistrome.shinyapps.io/timer). We also compiled the associations of 22 immune cell marker genes with C3AR1 mRNA levels in the TIMER, GEPIA and TCGA databases. The matrix score, immune score and ESTIMATE score between different C3AR1 expression groups are evaluated by the ESTIMATE algorithm. Infiltration level of immune cells in two C3AR1 groups was compared by the MCP counter algorithm. We further assessed the correlation between expression of C3AR1 and immune checkpoint.

### Correlation between C3AR1 expression and m6A modified genes

Enhancing or inhibiting m6A methyltransferase (writer) or demethylase (eraser) alters tumor development, and function status of m6A RNA methylation is highly dependent on the cell microenvironment. We analyzed gene expression of m6A mediators in two C3AR1 groups in the R software package. The prognostic value of C3AR1 related m6A genes in OC were explored. The ggplot2 is used for data visualization.

### Immunohistochemistry

Tissues of 5 OC patients and 5 healthy controls were stained by immunohistochemistry (IHC) after formalin fixation and paraffin-embedding. C3AR1 specific antibody (Proteintech) was diluted at 1:100.

### Cell culture, plasmid transfection and C3AR1 expression detection

Human ovarian cancer cell line SKOV3 was purchased from NEWGAINBIO (China). We maintained cells at 37 °C in a humidified atmosphere containing 5% carbon dioxide in RPMI 1640 medium (Gibco, USA) supplemented with 10% fetal bovine serum (Gibco, USA) and 1% penicillin/streptomycin solution (Gibco, USA). According to the manufacturer’s protocol, the plasmids (TSINGKE, China) with different concentrations were transfected into SKOV3 cells using jetPRIME transfection reagent (polyplus, China). After 48 h of transfection, Total RNA Extraction mini kit (Mabio,China) was used to extract total RNA from cultured cells for qRT-PCR. C3AR1 forward primer: CCCTACGGCAGGTTCCTATG; C3AR1 reverse primer: GACAGCGATCCAGGCTAATGG-3,‘ β-actin forward primer: GTGGGGCGCCCCAGGCACCAGGGC; β-actin reverse primer: CTCCTTAATGTCACGCACGATTTC. RIPA buffer (Solarbio) was used to lyse the cells and extract total proteins. Western blotting assay were conducted as described [17], the antibody concentration of C3AR1 (1:1000 ThermoFisher) and GAPDH (1:3000 Proteintech).

### EdU assay of cell proliferation rate

C3AR1 overexpression and control cells were treated with EdU reagent (Beyotime, C0075L) for 3 h. The cells were then immobilized with 4% paraformaldehyde and stained with fluorescent dye and Hoechst. Fluorescence detection was performed under an inverted fluorescence microscope.

### Statistical analysis

All data were statistically analyzed using GraphPad Prism (version 9.1.0). The student t test, one-way ANOVA, and Chi-square test were used to assess differences in variables between groups. The log-rank test evaluated the statistical significance of Kaplan-Meier survival curves. Spearman and statistical significance were used to analyze gene expression correlations. |R| > 0.3 is considered to be relevant, P values < 0.05 was considered statistically significant.

## Results

### Pan-cancer analysis of C3AR1 mRNA expression levels

**Fig. 1:**
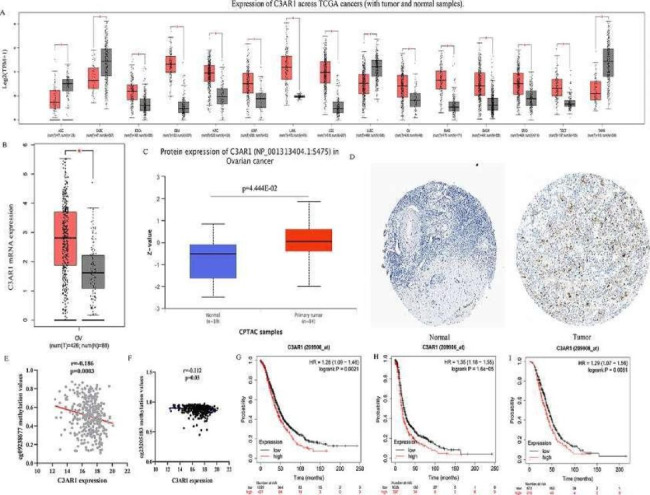
Analysis of the expression level of C3AR1 in pan-cancer. (A) The transcription level of C3AR1 in various cancer tissues in the GEPIA database. (B) C3AR1 mRNA expression levels in ovarian cancer and normal tissues in the GEPIA database. red (n = 426) tumor samples; gray (n = 88) normal samples. (C) C3AR1 protein levels in ovarian cancer and normal tissues in the CPTAC database. red (n = 84) tumor samples; blue (n = 19) normal samples. (D) The typical IHC staining of C3AR1 in ovarian cancer and normal tissues in the HPA database. (E, F) Correlation analysis between C3AR1 gene methylation levels (cg09238677, cg23205183) and transcription levels. Horizontal axis C3AR1 mRNA expression levels; vertical axis C3AR1 gene methylation levels. (G) OS analysis of C3AR1 in patients with ovarian cancer, (H) DFS and (I) PPS. DFS, disease-free survival; PPS, post progression survival; OS, overall survival. *P < 0.05; **P < 0.01; *** P < 0.001; ****P < 0.0001

First, we compared the difference of C3AR1 between ovarian cancer and other human tumors and normal tissues. Increased expression of C3AR1 is higher in esophageal carcinoma (ESCA), glioblastoma multiforme (GBM), kidney renal clear cell carcinoma (KIRC), kidney renal papillary cell carcinoma (KIRP), acute myeloid leukemia (LAML), brain lower grade glioma (LGG), ovarian serous cystadenocarcinoma (OC), pancreatic adenocarcinoma (PAAD), skin cutaneous melanoma (SKCM), stomach adenocarcinoma (STAD), and testicular germ cell tumors (TGCT) than normal tissues. However, C3AR1 mRNA expression levels in adrenocortical carcinoma (ACC), lymphoid neoplasm diffuse large B-cell lymphoma (DLBC), lung squamous cell carcinoma (LUSC), and thymoma (THYM) are lower than normal tissues (Fig. 1A).

### Increased C3AR1 is associated with poor prognosis of OC patients

We investigated mRNA and protein expression of C3AR1 in the OC and normal cohorts in the GEPIA, CPTAC and HPA datasets. Results showed that C3AR1 mRNA in OC patients was about twice that of the normal group (Fig. 1B). In addition, compared with non-metastatic cells ES-2 and OV56, metastatic cells Hey-A8, ONCO-DG-1, and 59 M have higher C3AR1 mRNA levels (Figure S1). To further verify the results, C3AR1 protein levels in OC and normal samples were analyzed. As shown in Fig. 1C and D, C3AR1 protein levels in tumor sample tissues were higher than that in normal tissues, which were mainly distributed in the membrane and cytoplasm of cancer cells. Immunohistochemical results from our cohort showed that C3AR1 expression was higher in the ovarian cancer group than in the normal group (Figure S1A).In the TCGA OC HumanMethylation450K cohort, transcription levels of C3AR1 in OC patients were negatively correlated with its DNA methylation levels (cg09238677, r=-0.186, p < 0.05; cg23205183, r=-0.112, p < 0.05.) (Fig. 1E, F). We further evaluated the prognostic potential of C3AR1 in OC. The overall survival (HR = 1.26, 95%CI (1.09–1.46), P = 0.0021), disease-free survival (HR = 1.35, 95%CI (1.18–1.55), P = 1.6E-05), post progression survival (HR = 1.29, 95%CI (1.07–1.56), P = 0.0081) (Fig. 1G-I) were worse in OC patients with higher C3AR1 expression levels (Fig. 1G-I).

### Increased C3AR1 correlated with malignant clinicopathological features and poor prognosis of patients with OC

Relationships between C3AR1 and clinicopathological features were analyzed in OC patients in the TCGA. Results showed that elevated C3AR1 was significantly related to multiple malignant characteristics, including tumor grade, stage, days to new tumor event, lymph node metastasis, cancer status, and survival status (Table 1). However, little differences of C3AR1 were observrd in different ages and anatomic neoplasm subdivision. Crucially, C3AR1 is positively correlated with 18 tumor metastasis related genes including FN1, SNAI2, and ZEB2 (Figure S2).

**Table 1:** Correlations between C3AR1 and clinicopathological parameters of ovarian cancer patients in the TCGA

Catagory		H group	L group	P-value
Age	< 60	152	148	0.76
	> 60	127	130	
Anatomic neoplasm subdivision	single	68	76	0.35
	bilateral	197	183	
Stage	III-IV	265	244	0.0004
	I-II	11	34	
Days to new tumor event	> 1 year	97	132	< 0.0001
	< 1 year	76	39	
Lymphatic invasion	Y	91	44	0.0013
	N	35	43	
Neoplasm histologic grade	G3	238	211	0.02
	G2	33	50	
New neoplasm event	recurrence	142	123	0.09
	progression	9	16	
Cancer status	tumor free	44	71	0.0043
	with tumor	201	175	
Vital status	deceased	123	105	0.0047
	living	155	172	
Additional radiation therapy	Y	22	8	0.03
	N	139	125	

H high C3AR1 expression; L low C3AR1 expression; Y yes; N no

### Screening and functional enrichment analysis of C3AR1 co-expressed genes in OC

**Fig. 2:**
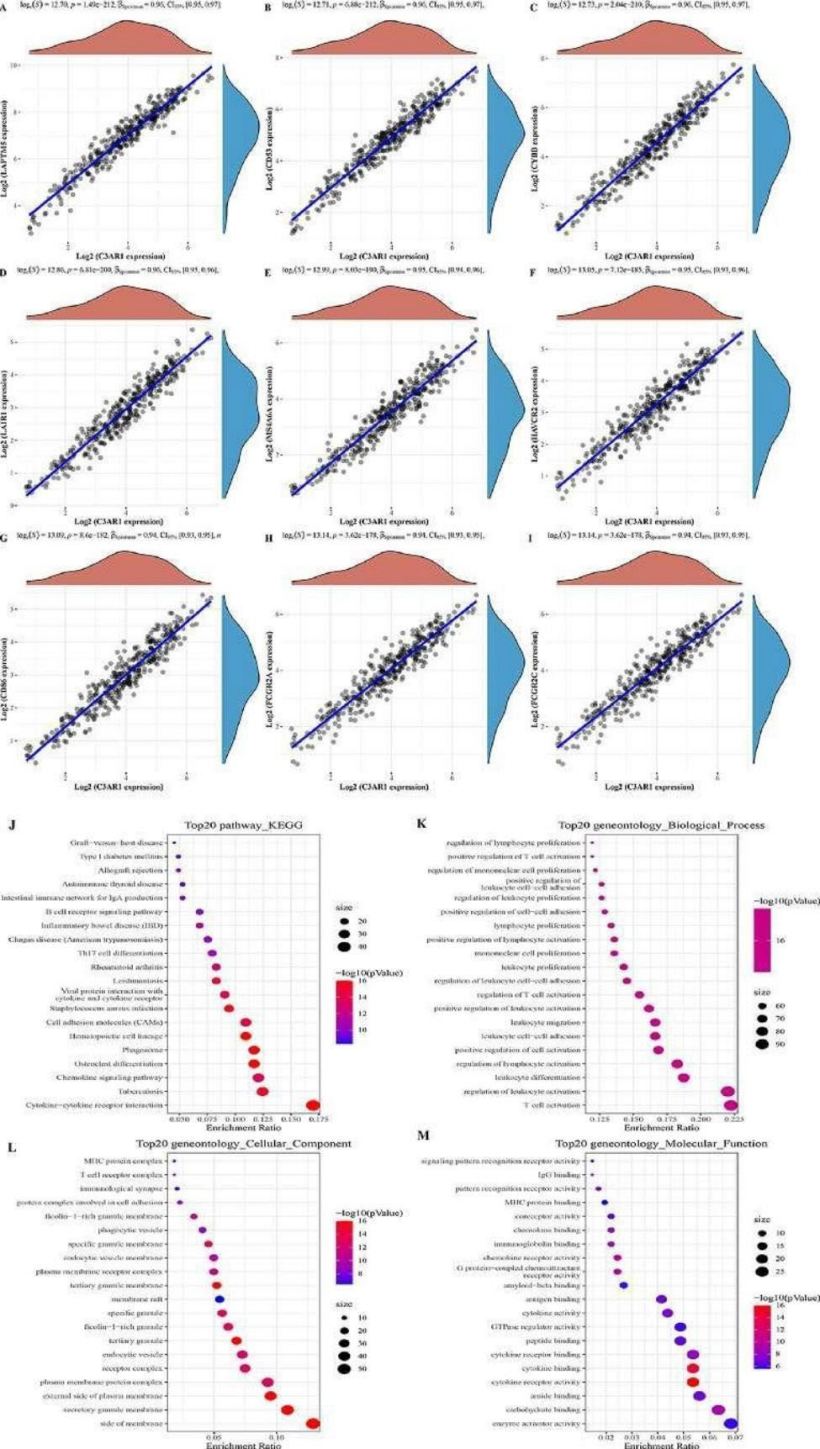
Functional and pathway enrichment analysis of C3AR1 co-expressed genes. (A) Spearman correlation analysis of C3AR1 and LAPTM5 transcription levels. (B) C3AR1 and CD53. (C) C3AR1 and CYBB. (D) C3AR1 and LAIR1. (E) C3AR1 and MS4A6A. (F) C3AR1 and HAVCR2. (G) C3AR1 and CD86 cause. (H) C3AR1 and FCCR2A. (I) C3AR1 and FCCR2C. The horizontal axis and the vertical axis each represent the expression distribution of a gene, and the curve represents the trend of the respective gene distribution; the correlation p value, correlation coefficient and correlation calculation method are placed on the top side. (J) Top 20 KEGG signaling pathways enriched. (K) Top 20 biological processes enriched of C3AR1 co-expressed genes. (L) Top 20 cellular components enriched. (M) Top 20 molecular function enriched. Dot size indicates number of related genes, and color intensity indicates the significance of enrichment

Co-expressed genes of C3AR1 were analyzed by R package and visualized through the ggplot package. 11,967 co-expressed genes were identified (8123 positive correlations, 3844 negative correlations, P < 0.05)). Top 9 genes with the strongest correlation with C3AR1 are presented (Fig. 2A-I), including LAPTM5 (cor = 0.96, P = 1.49E-212), CD53 (cor = 0.96, P = 6.88E-212), CYBB (cor = 0.96, P = 2.04E-210), LAIR1 (cor = 0.96, P = 6.81E-200), MS4A6A (cor = 0.95, P = 8.03E-190), HAVCR2 (cor = 0.95, P = 7.12 E-185), CD86 (cor = 0.94, P = 8.6E-182), FCCR2A (cor = 0.94, P = 3.62E-178) and FCCR2C (cor = 0.94, P = 3.62E-178). Co-expressed genes with coefficient |cor|> 0.6, P < 0.05 were analyzed by GO (gene ontology) and KEGG pathway enrichment analysis and enriched in 945 biological processes (GO-BP), 80 cell components (GO-CC), 68 molecular functions (GO-MF) and 61 KEGG signaling pathways. GO analysis showed that these genes are mainly involved in T cell, leukocyte and lymphocyte activation and cytokine and chemokine activity (Fig. 2K-M). KEGG pathway analysis showed that co-expressed genes are mainly involved in cytokine-cytokine receptor interaction, tuberculosis, and chemokine signaling pathways (Fig. 2J).

### Identification and functional enrichment analysis of differentially expressed genes in patients with different C3AR1 transcription levels in OC

**Fig. 3:**
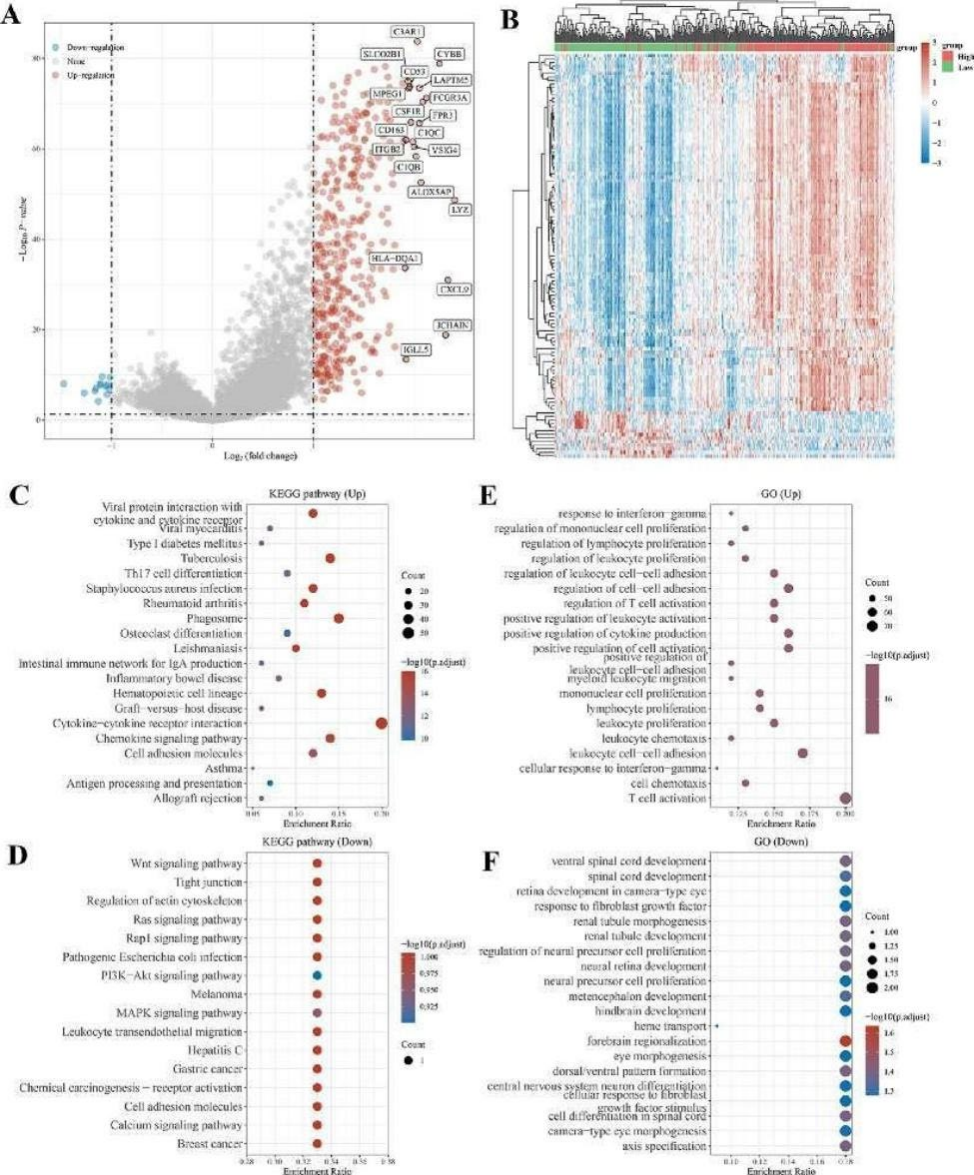
Screening and enrichment analysis of DEGs of C3AR1. (A) The volcano plot shows DEGs of C3AR1 high vs. low expression group. Red represents up-regulated genes and green represents down-regulated genes. The genes with the most significant differences are shown in the figure. (B) The cluster plot shows DEGs of C3AR1 high expression vs. low expression group. The abscissa represents the patient, the ordinate represents the gene, and the gene expression level is marked by different colors. (C) and (D) KEGG analysis of pathways related to DEGs. (E) and (F) Gene ontology analysis of biological processes negatively related to DEGs. The size of the dot represents the number of differentially expressed genes, and the intensity of the color indicates the significance of the enrichment result

To further characterize the molecular function of C3AR1, patients with ovarian cancer in TCGA were devided into high and low C3AR1 groups. 415 DEGs (402 up-regulated and 13 down-regulated) were presented in the volcano plot (Fig. 3A). Distinct different gene expression patterns between the two groups were visualized in the cluster plot (Fig. 3B). KEGG analysis was performed regarding DEGs, and 69 up-regulated and 2 down-regulated pathways were found, among which cytokine-cytokine receptor interaction (FDR = 4.17E-24, P = 4.18E-24), the hematopoietic cell lineage (FDR = 1.08E-23, P = 4.18E-24) and the chemokine signaling pathway (FDR = 9.25E-16, P = 3.94E-17) are the most significantly enriched (Fig. 3C-D). Gene ontology analysis revealed 1135 up-regulated biological processes and 156 down-regulated biological processes, among which T cell activation (FDR = 2.49E-41, P = 7.02E-45) and leukocyte cell-cell adhesion (FDR = 8.32E-44, P = 1.47E-40) and leukocyte proliferation (FDR = 5.18E-39, P = 6.12E-36) are the most significantly enriched (Fig. 3E-F). We also applied the ESTIMATE algorithm to calculate stromal score, immune score, and ESTIMATE score. The higher C3AR1 levels is associated with higher the immune score, stromal score and ESTIMATE score (Figure S3). The enrichment analysis of DEGs and co-expressed genes of C3AR1 are highly consistent, indicating that C3AR1 may be related to tumor immune cells or immune regulation.

### Correlation analysis between C3AR1 and tumor immune cell infiltration

**Fig. 4:**
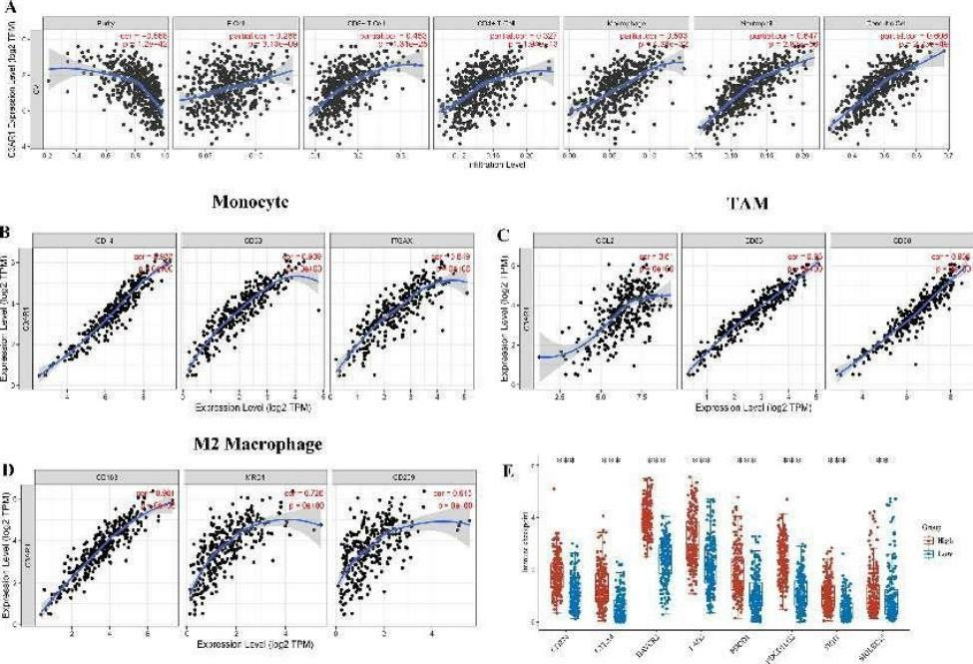
Correlation between C3AR1 and OC tumor immune infiltration. (A) Correlation between C3AR1 expression and immune cell infiltration in OC in TIMER database. (B-D) C3AR1 expression is associated with M2 macrophages, tumor-associated macrophages and monocyte immune marker genes in ovarian cancer. Markers for M2 macrophages: CD163, MRC1, CD209, tumor-associated macrophages: CCL2, CD86, CD68, and monocytes: CD14, CD33, ITGAX. (E) Gene expression levels of 8 common immune checkpoint genes in the C3AR1 high and low expression group. ** p < 0.01,*** p < 0.001

Correlation between C3AR1 expression in OC and immune cell infiltration were evaluated and found that C3AR1 is positively correlated with the six types of immune cells. According to correlation index, they are neotrophil (r = 0.647, P = 2.83E-58), dendritic cell (r = 0.606, P = 2.23E-49), macrophage (r = 0.503, P = 4.36E-32), CD8 + T cells (r = 0.453, P = 1.31E-32), CD4 + T cells (r = 0.327, P = 1.94E-13) cells and B cells (r = 0.266, P = 3.13E-09) (Fig. 4A). MCP counter analysis showed that patients have higher C3AR1 tend to have more tumor immune cell infiltration (Figure S4), including T cells, cytotoxic lymphocytes, B lineage, NK cells, monocytic lineage, myeloid dendritic cells, neutrophils, endothelial cells and fibroblasts (P < 0.01).

We further evaluated the association between C3AR1 and various immune cell markers in OC cohorts of TIMER, GEPIA and TCGA database (Table 2). Results show that C3AR1 has the strongest correlation with M2 macrophages (CD163, MRC1, CD209), tumor-associated macrophages (CCL2, CD86, CD68) and monocyte immune markers, including (CD14, CD33, ITGAX) (Fig. 4B-D). In addition, we evaluated the interaction between C3AR1 and 22 immune checkpoint genes. As shown in Fig. 4E, the mRNA of 8 immune checkpoints (CD274, CTLA4, HAVCR2, LAG3, PDCD1, PDCD1LG2, TIGIT, SIGIEC15) increased significantly in the C3AR1 high group. Therefore, these evidences indicate that C3AR1 plays an important role in immune tolerance and immune escape of OC cells.

**Table 2:** Correlation of C3AR1 with immune cell biomarkers in TIMER, GEPIA, and TCGA databases

Description	Gene markers	TIMER		GEPIA		TCGA	
		Cor	p	Cor	p	Cor	p
B cell	CD19	0.10	9.79E-02	-0.02	0.68	0.07	1.98E-01
	MS4A1	0.55	5.07E-25	0.19	8.70E-05	0.35	4.91E-12
	CD79A	0.42	2.13E-14	0.22	4.00E-06	0.37	7.88E-14
CD8 + T Cell	CD8A	0.63	0.00E + 00	0.50	0.00E + 00	0.63	3.70E-42
	CD8B	0.48	0.00E + 00	-0.01	0.98	0.33	7.64E-11
	IL2RA	0.76	0.00E + 00	0.68	0.00E + 00	0.77	3.41E-74
Tfh	CXCR3	0.69	0.00E + 00	0.51	0.00E + 00	0.70	1.33E-56
	CXCR5	0.42	1.83E-14	-0.06	0.22	0.27	9.07E-08
	ICOS	0.65	4.04E-38	0.70	6.40E-67	0.64	5.78E-45
Th1	IL12RB1	0.83	2.03E-79	0.85	7.40E-122	0.79	3.67E-80
	CCR1	0.92	0.00E + 00	0.89	1.50E-146	0.88	1.11E-119
	CCR5	0.88	2.03E-100	0.89	4.10E-143	0.86	6.12E-111
Th2	CCR4	0.69	4.34E-44	0.70	3.40E-65	0.64	1.43E-44
	CCR8	0.51	3.61E-21	0.54	5.50E-33	0.33	3.63E-11
	HAVCR1	0.18	1.66E-03	0.31	7.50E-11	-0.01	9.15E-01
Th17	IL21R	0.79	5.63E-67	0.82	6.50E-103	0.75	1.52E-69
	IL23R	0.07	2.24E-01	0.15	1.70E-03	0.10	5.05E-02
	CCR6	0.44	0.00E + 00	0.56	3.10E-37	0.39	5.93E-15
Treg	FOXP3	0.61	0.00E + 00	0.64	4.30E-50	0.62	1.59E-40
	NT5E	0.47	0.00E + 00	0.51	4.00E-29	0.47	2.95E-22
	IL7R	0.69	0.00E + 00	0.71	1.50E-67	0.67	7.68E-51
T cell exhaustion	PDCD1	0.54	2.20E-24	0.56	8.70E-37	0.53	5.05E-28
	CTLA4	0.65	2.05E-38	0.69	1.10E-60	0.63	3.44E-43
	LAG3	0.58	0.00E + 00	0.52	7.20E-31	0.55	1.21E-30
M1 Macrophage	NOS2	0.06	2.99E-01	0.23	1.40E-06	0.07	1.61E-01
	IRF5	0.42	4.76E-15	0.47	2.30E-24	0.48	1.33E-22
	PTGS2	0.23	3.98E-05	0.30	2.60E-10	0.24	2.34E-06
M2 Macrophage	CD163	0.90	0.00E + 00	0.82	1.40E-103	0.91	3.06E-146
	MRC1	0.73	0.00E + 00	0.78	7.10E-88	0.72	2.55E-61
	CD209	0.61	0.00E + 00	0.70	2.00E-64	0.62	4.07E-41
TAM	CCL2	0.61	0.00E + 00	0.59	4.20E-42	0.63	5.97E-42
	CD86	0.95	0.00E + 00	0.95	1.20E-223	0.95	2.34E-191
	CD68	0.94	0.00E + 00	0.93	9.80E-181	0.72	4.95E-62
Monocyte	CD14	0.93	0.00E + 00	0.93	7.90E-193	0.94	2.90E-170
	CD33	0.91	0.00E + 00	0.88	1.00E-138	0.88	3.49E-125
	ITGAX	0.85	0.00E + 00	0.84	8.60E-117	0.85	2.15E-103
Natural killer cell	B3GAT1	0.08	1.87E-01	0.20	4.70E-05	-0.04	4.96E-01
	KIR3DL1	0.33	2.49E-09	0.45	8.20E-23	0.35	4.27E-12
	CD7	0.61	0.00E + 00	0.63	1.30E-47	0.60	1.76E-38
Neutrophil	FCGR3A	0.92	4.84E-126	0.94	6.20E-195	0.94	4.66E-170
	CD55	0.20	4.58E-02	0.34	6.10E-13	0.28	4.80E-08
	ITGAM	0.89	0.00E + 00	0.92	8.10E-171	0.91	8.75E-142
Dendritic cell	CD1C	0.53	6.36E-23	0.57	2.40E-37	0.50	6.45E-25
	THBD	0.55	0.00E + 00	0.64	2.40E-50	0.57	2.69E-34
	NRP1	0.51	3.54E-21	0.54	1.10E-33	0.53	7.75E-29

**Fig. 5:**
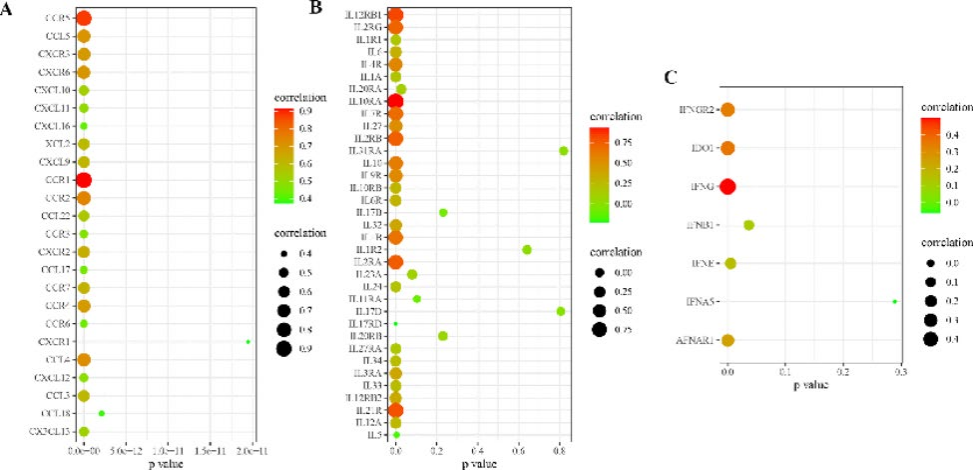
Correlation analysis between C3AR1 and cytokines in the tumor microenvironment, including chemokines and their receptors (A), interleukins and their receptors (B), and interferons and their receptors (C). The larger the point, the darker the color, the stronger the correlation

Cor correlation coefficient; TAM, tumor-associated macrophages; Th1/2, T helper type 1/2 cells; Treg, regulatory T cells; P, P-value;

Enrichment analysis of co-expressed genes and DEGs indicated that C3AR1 is involved in the densely mediated signaling pathways of chemokines, interleukins, and interferons. Therefore, we evaluated the correlation between C3AR1 and chemokines, interleukins and interferons in the ovarian cancer microenvironment in the TCGA database. As shown in Fig. 5, C3AR1 has strong correlations with 24 chemokines (Fig. 5A) including CCR1 and CCR5, 26 interleukins (Fig. 5B) including IL2RA, IL10RA and IL21R, and six interferons (Fig. 5C) including IDO1 and INFG. All these factors have been shown to be important immune regulators.

### Correlation between C3AR1 and m6A modifiers in OC

M6A-related genes are widely involved in multiple processes such as ovarian cancer metastasis, chemotherapy resistance, and tumor microenvironment regulation. Analysis of TCGA found that C3AR1 co-expressed with multiple m6A-related genes, including HNRNPC, IGF2BP2, RBMX and ZC3H13, which were significantly positively correlated (Figure S5A, P < 0.05). Genes significantly negatively correlated with C3AR1 include ALKBH5, IGFBP3, METL14, RBM15, WTAP, YTHDC2 and YTHDF3 (P < 0.05). In addition, these 9 methylation related genes were differentially expressed in C3AR1 high expression and low expression groups (RBMX, IGFBP2, WTAP, VIRMA, YTHDF3, METTL14, FTO, HNRNPC and YTHDC2) (Figure S6). Kaplan-Meier curve shows FTO (HR = 1.26, 95%CI 1.1–1.44, P = 0.001), IGFBP3 (HR = 1.25, 95%CI 1.08–1.44, P = 0.0031), WTAP (HR = 1.39, 95%CI The high expression of 1.21–1.59, P = 1.5E-06) and YTHDF3 (HR = 1.18, 95%CI 1.03–1.34, P = 0.015) are closely related to the poor prognosis of LUAD (Figure S5B-E). These results indicate that C3AR1 ultimately affects the progression and prognosis of ovarian cancer by participating in the m6A modification of OC, especially with WTAP.

### C3AR1 promotes the proliferation of ovarian cancer cells

**Fig. 6:**
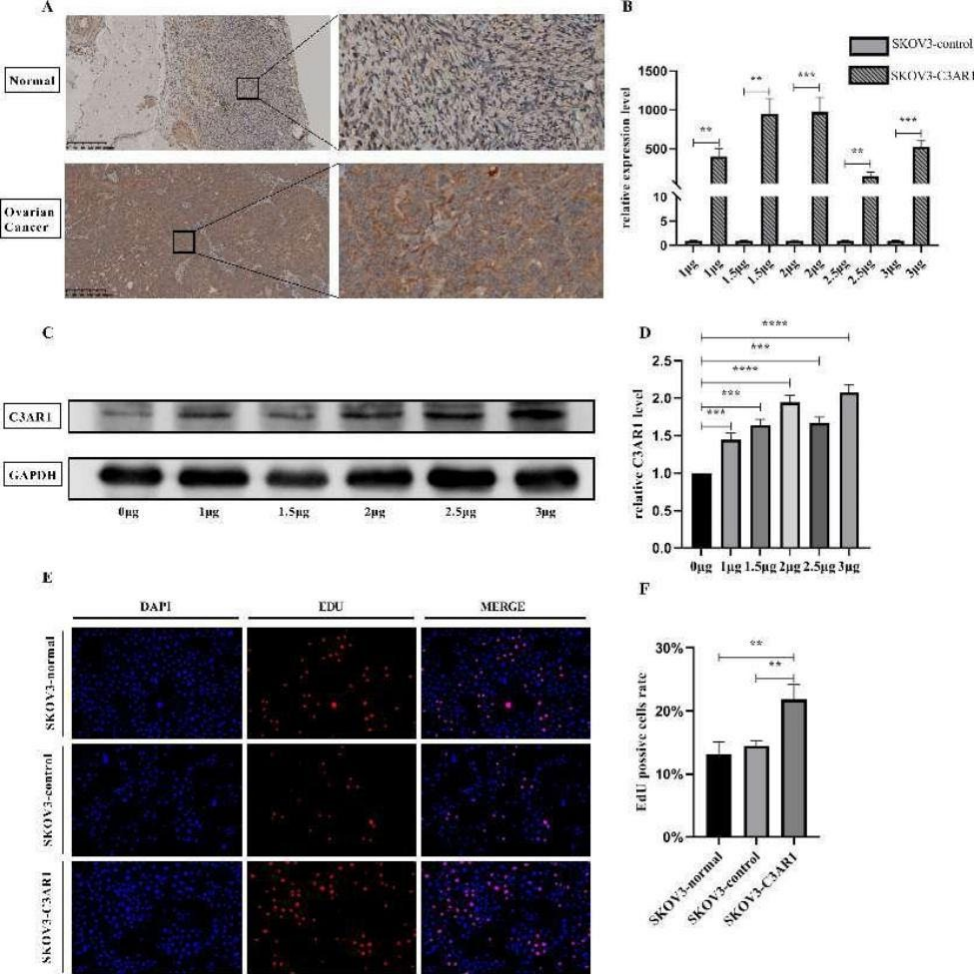
C3AR1 promotes the proliferation of ovarian cancer cells. (A) the expression of C3AR1 in ovarian carcinoma was higher than that in normal ovarian tissue. The scale bar is at the bottom left of the picture. (B) The expression level of C3AR1 detected by RT-qPCR at 48 h after different concentration of plasmid transfection. (C-D) The expression and Quantitative analysis of C3AR1 protein were analyzed by Western blot after 48 h of plasmid transfection. (E-F) The cell proliferation of SKOV3 group, empty plasmid group and over-expression group was detected and quantified by Edu staining

The expression level of C3AR1 in SKOV3 cells was low, therefore we overexpressed C3AR1 to conduct subsequent functional experiments. mRNA and protein levels of C3AR1 were significantly increased after plasmid transfection (Fig. 6B-D), and the mRNA overexpression efficiency was the highest when the plasmid was 2ug/ml. Therefore, 2ug/ml plasmid was selected for subsequent study. The cell proliferation rate of the control group, empty vector plasmid group and C3AR1 plasmid group was detected by EdU assay. It was found that C3AR1 overexpression significantly promoted the proliferation of SKOV3 cells (Fig. 6E).

## Discussion

In this study, the expression of C3AR1 in OC was analyzed and verified from both mRNA and protein levels by bioinformatics and experimental methods. Correlation analysis between the methylation and the expression of C3AR1 showed that low methylation levels partly contribute to the high C3AR1 mRNA level. Previous studies have found that GBM, LGG, and COAD patients with high C3AR1 expression tend to have a poor [14].We also confirmed that the prognosis (OS, PFS, PPS) of OC patients with high C3AR1 expression is poor. Morever, it was found that C3AR1 expression was related to tumor grade, stage, recurrence, lymph node metastasis and survival status. In vitro experiment indicates that increased C3AR1 promotes the proliferation of ovarian cancer cells. These evidence suggests that C3AR1 can be used as a potential prognosis biomarker for evaluating the prognosis of OC patients and targeting C3AR1 may help improve the treatment of OC patients.

Current researches on C3AR1 in tumors mainly focuses on stomach adenocarcinomas (STAD), [18] and kidney renal clear cell carcinoma (KIRC)[19]. There are few studies regarding the biological and molecular functions of C3AR1 in OC.

We analyzed the related genes of C3AR1 and found that the expression of LAPTM5, CD53, CYBB, LAIR1, MS4A6A, HAVCR2, CD86, FCCR2A and FCCR2C in OC has the strongest correlation with C3AR1. Chen et al. found that down-regulation of LAPTM5 induces cell cycle arrest in G0/G1 phase to inhibit the proliferation and viability of bladder cancer [20]. HAVCR2 inhibits anti-tumor immunity by interacting with its ligand Galectin 9 (Gal9), phosphatidylserine (Ptdser) high mobility group box 1 (HMGB1), and carcinoembryonic antigen-related cell adhesion molecule 1 (CEACAM1)[21]. GO and KEGG analysis of C3AR1 co-expressed genes showed that C3AR1 was mainly correlates with T cell, leukocyte and lymphocyte activation and cytokine and chemokine activity. KEGG pathway analysis showed that C3AR1 was mainly related to cytokine-cytokine receptor interaction and chemokine signaling pathway. Enrichment analysis results of DEGs confirmed this result again. A number of studies have shown that multiple chemokines and cytokines are involved in the progression of ovarian [22, 23].

Immune cell infiltration affects tumor metastasis, chemotherapy resistance, and prognosis of OC patients. We analyzed data from multiple databases and confirmed that C3AR1 is closely related to immune cell infiltration, including macrophages. In addition, immune cell markers from 3 data sets also confirmed these results. Among them, C3AR1 has the strongest correlation with TAM, M2 macrophage and monocyte markers, suggesting that C3AR1 may affect the immune microenvironment of OC mainly by regulating macrophage infiltration. The role of M2 and TAM in promoting tumor progression in ovarian cancer has been widely recognized. In ovarian cancer, M2 macrophages promote the formation of ascites by reducing the expression of VLA4 in their cell membranes and reducing the level of VCAM1 in endothelial [24]. Mechanistically speaking, the down-regulation of VLA4 or VCAM1 results in the inhibition of the RAC1 / ROS / PYK2 (p-PYK2) /p-VE-cad cascade, thereby enhancing cell adhesion. In addition, targeting the VLA4/VCAM1 axis can enhance the vascular barrier and inhibit the formation of ascites in the body. At the same time, studies have found that EGF derived from TAMs promoting the early transluminal metastasis. Specifically, TAM in ovarian cancer activates EGFR through secreted EGF and up-regulates VEGF/VEGFR signals in peripheral tumor cells, supporting tumor cell proliferation and migration. Blocking EGFR or ICAM-1 in TAM by drugs or antibodies inhibits tumor tissue formation and disease progression in mouse models of ovarian [25]. We speculate that the overexpression of C3AR1 promotes the infiltration of TAM cells and M2 macrophages in OC, thereby accelerating tumor progression. Finally, we found that 8 immune checkpoints including CD274 (PD-1) are closely related to C3AR1. Patients with OC who respond to immune checkpoint blockade treatment will have long-term benefits. An anti-PD-1/PD-L1 therapy’s efficacy is partly determined by the expression of PD-L1 on tumour cells. Compared with patients expressing low PD-L1, those with high PD-L1 expression have higher response rate. This can be attribute to the strong immunosuppression and low mutation burden in tumor microenvironment. Several studies have suggested defective DNA repair machinery is associated with higher levels of neoantigens in OC patients with BRCA mutations and homologous recombination defects. The most effective immunotherapy relies in newly diagnosed OC and overcome the exhausted immune system. Several strategies have been develop to enhance sensitivity to immunotherapy in OC, including the dual immune checkpoint blockade, as well as a combination therapies involving immune checkpoint and PARP inhibitors (PARPi), cytotoxic drugs, radiotherapy, and/or angiogenesis [26]. Studies have shown that CDK4/6 inhibition combined with PD-1 blockade treatment has higher CXCL10 and CXCL13 levels and CD8 + and CD4 + T cell activity than monotherapy-treated ovarian cancer [27]. We speculate that the combined use of small molecule inhibitors targeting C3AR1 and ICB may be a promising strategy for the treatment of ovarian cancer. However, our hypothesis on the relationship between C3AR1 and macrophage infiltration and immune checkpoint blocking therapy still needs more research evidence to verify.

Several studies have confirmed m6A mediators in promoting ovarian cancer proliferation, angiogenesis, chemotherapy resistance and tumor microenvironment regulation. Wang et al. found that knocking down YTHDF1 in cisplatin-resistant ovarian cancer cells can inhibit cancer stem cell-like characteristics by overexpression of TRIM29 [28]. Liu et al. confirmed that YTHDF1 is up-regulated in high-grade serous ovarian cancer and is related to tumor grade, FIGO staging, and overall [29]. And through multi-omics studies, it is determined that its direct target in ovarian cancer cells is the translation initiation factor EIF3C, and YTHDF1 controls the translation of EIF3C in a m6A-dependent manner. In addition, many m6A-related factors such as YTHDF2, LETM1, METL3 and ALKBH5 have also been confirmed to be associated with the malignancy of ovarian cancer. This study found that C3AR1 was significantly correlated with ALKBH5, IGFBP3, METL14, RBM15, WTAP, YTHDC2, and YTHDF3. It was also revealed that the expression levels of RBMX, IGFBP2, WTAP, VIRMA, YTHDF3, METL14, FTO, HNRNPC, and YTHDC2 in the C3AR1 high expression group were significant changed. Finally, survival curve analysis shows that patients with ovarian cancer with high WTAP expression have a worse prognosis. We speculate that C3AR1 may be involved in m6A-related gene-mediated tumor regulation, and its tumor-promoting effect may be achieved through the modulation of WTAP.

This study demonstrates that C3AR1 is up-regulated and promotes cancer cell proliferation in ovarian cancer, as well as associated with various pathological features. In particular, C3AR1 overexpression is associated with immune cell infiltration, mediating the tumor immune microenvironment, and is associated with poor prognosis of ovarian cancer patients. Patients with high C3AR1 are more likely to benefit from immunotherapy as a result of elevated immune checkpoints expression levels. Taken together, these results indicate that C3AR1 may be a potential new immunotherapeutic target for ovarian cancer.

Authors’ contributions.

Kaixian Deng conceptualized the study design and supervised the analysis. Jinfa Huang collected the data, performed the statistical analysis, and wrote the paper. Lei Zhou conducted the experiment and polished the draft. All authors read and approved the final manuscript.

## Electronic supplementary material

Below is the link to the electronic supplementary material.

Supplementary Material 1

Supplementary Material 2

Supplementary Material 3

Supplementary Material 4

Supplementary Material 5

Supplementary Material 6

## Data Availability

The authors declare that the data and material of this study are available within the article.
