# Supplementary figures and images for: Prognostic marker C3AR1 is associated with ovarian cancer cell proliferation and immunosuppression in the tumor microenvironment
Source: J Ovarian Res. 2023 Apr 1;16:64. doi: 10.1186/s13048-023-01140-2 (PMC10067206; doi:10.1186/s13048-023-01140-2)

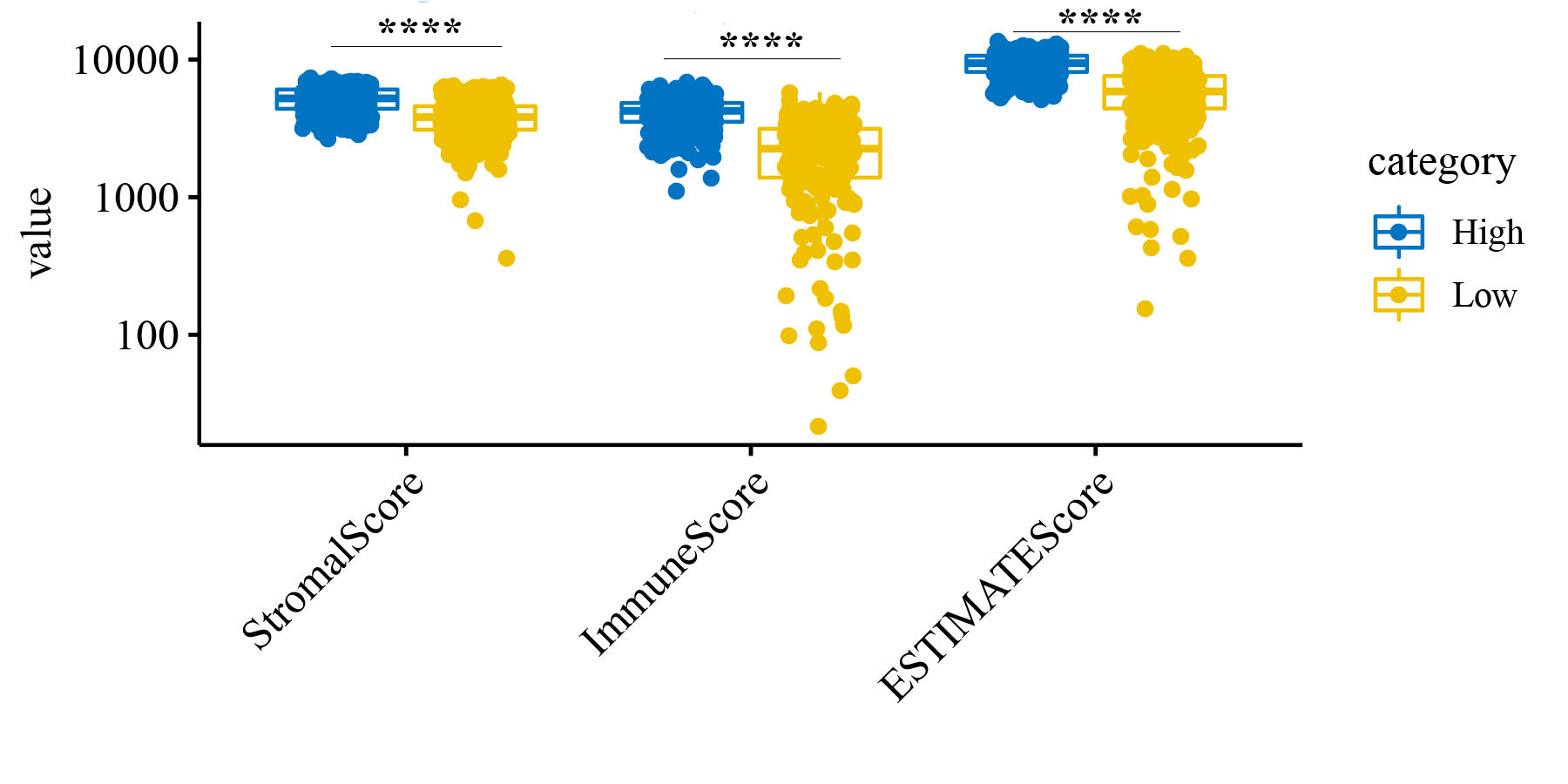

Supplement: Supplementary file 1 — Supplementary Material 1 [file 13048_2023_1140_MOESM1_ESM.tif]

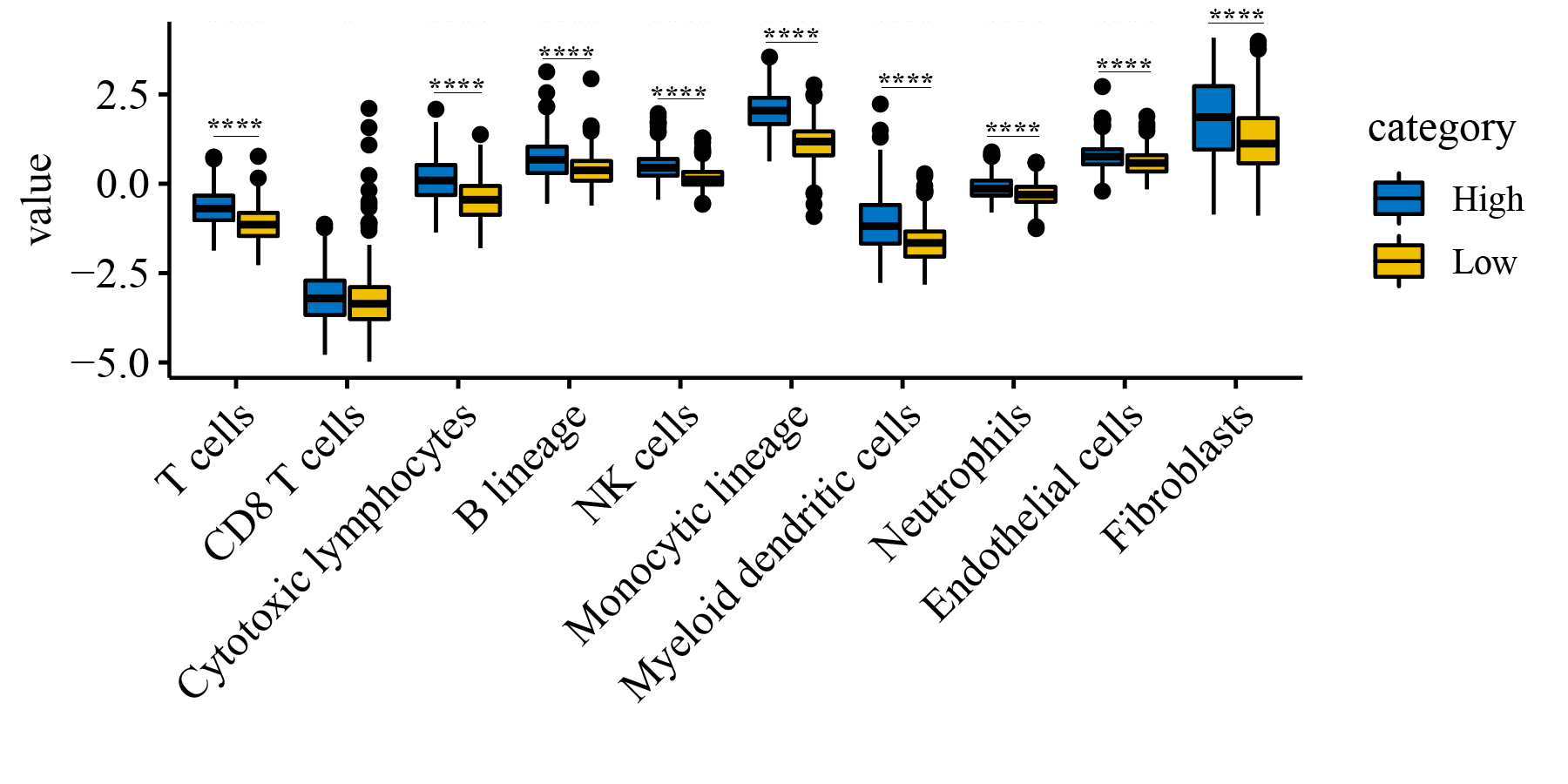

Supplement: Supplementary file 2 — Supplementary Material 2 [file 13048_2023_1140_MOESM2_ESM.tif]

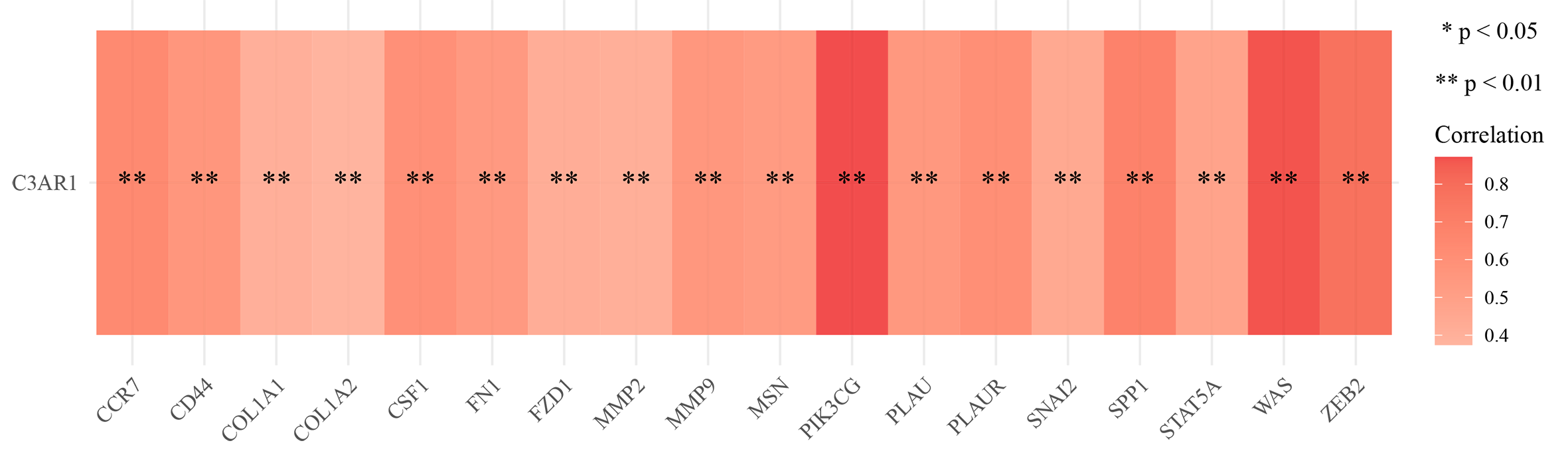

Supplement: Supplementary file 3 — Supplementary Material 3 [file 13048_2023_1140_MOESM3_ESM.tif]

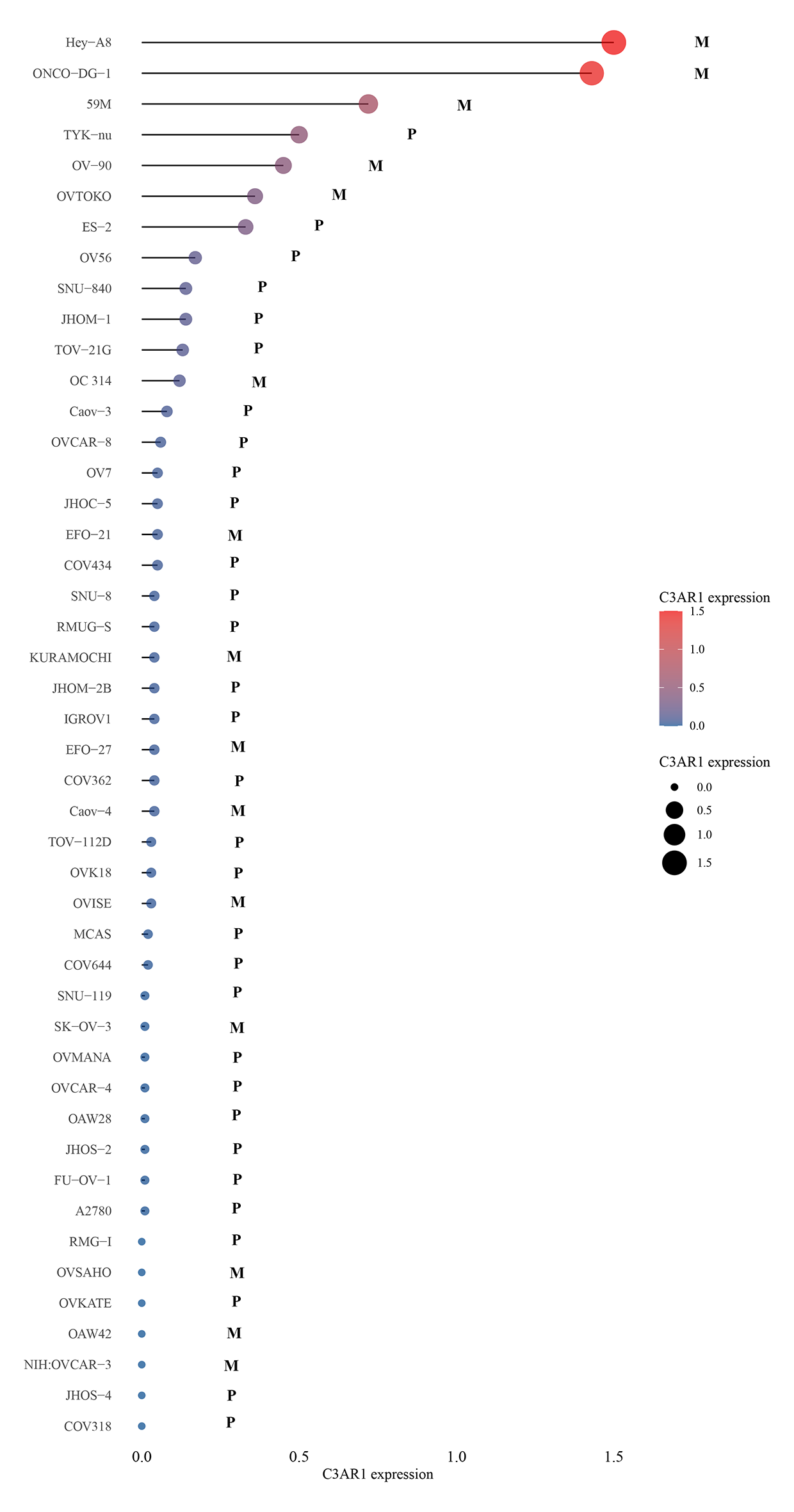

Supplement: Supplementary file 4 — Supplementary Material 4 [file 13048_2023_1140_MOESM4_ESM.tif]

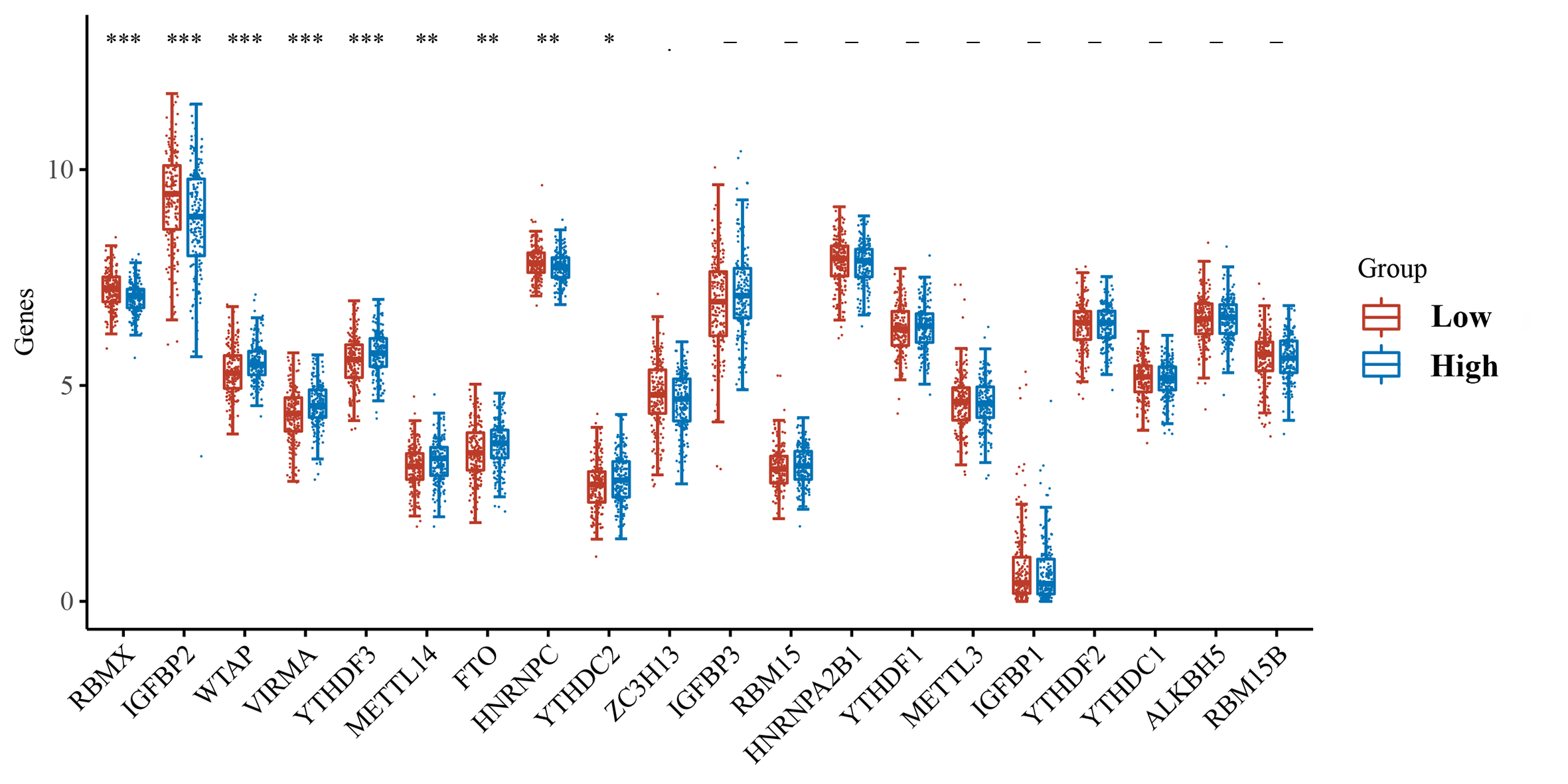

Supplement: Supplementary file 5 — Supplementary Material 5 [file 13048_2023_1140_MOESM5_ESM.tif]

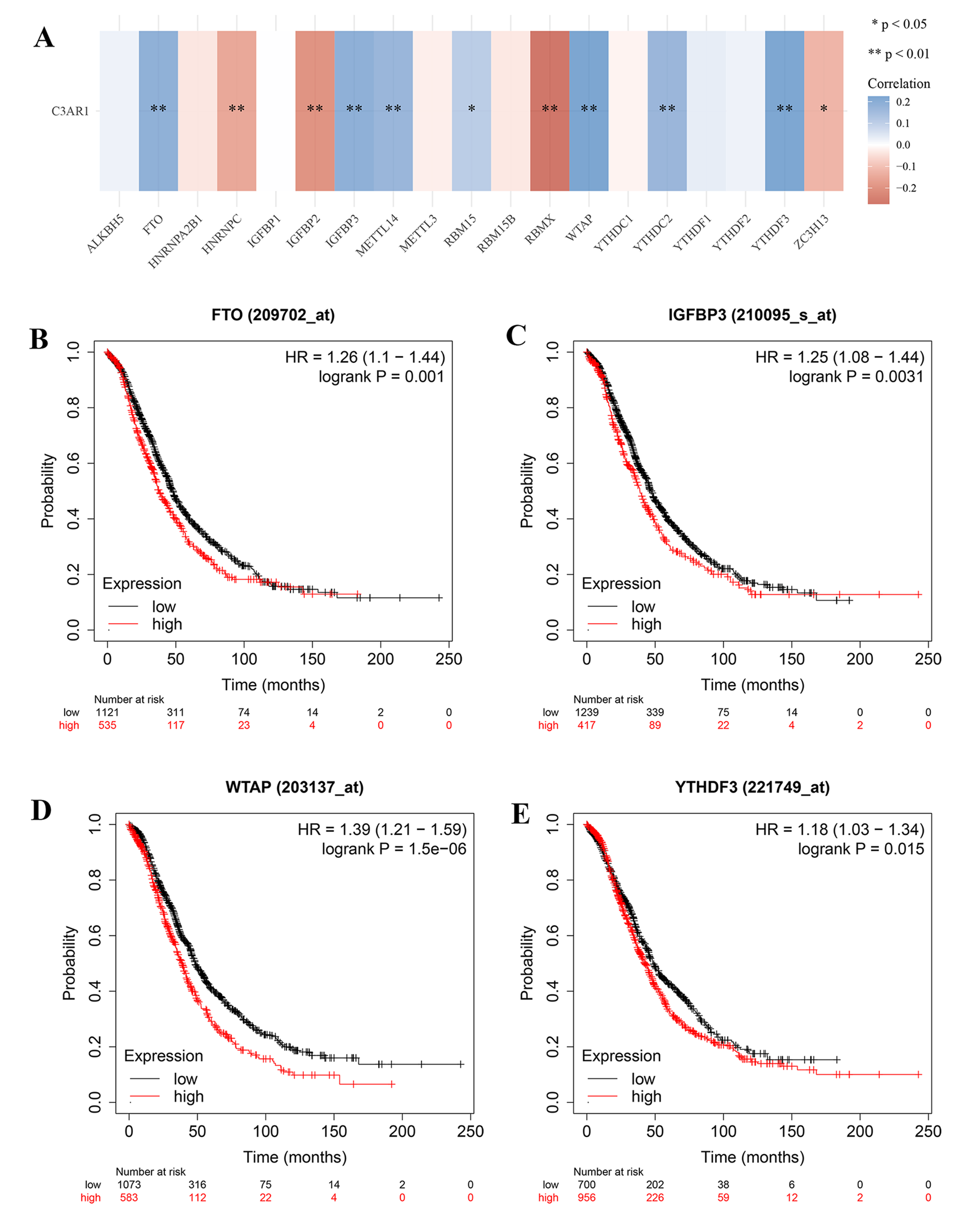

Supplement: Supplementary file 6 — Supplementary Material 6 [file 13048_2023_1140_MOESM6_ESM.tif]
